# Eight decades of research on the long-term health effects of radiation in atomic bomb survivors and their offspring

**DOI:** 10.1093/carcin/bgaf047

**Published:** 2025-10-27

**Authors:** Kenji Kamiya, Ritsu Sakata, Preetha Rajaraman

**Affiliations:** Executive Director, Radiation Effects Research Foundation, 5-2 Hijiyama Park, Minami-ku, Hiroshima 732-0815, Japan; Department of Epidemiology, Radiation Effects Research Foundation, 5-2 Hijiyama Park, Minami-ku, Hiroshima 732-0815, Japan; Executive Director, Radiation Effects Research Foundation, 5-2 Hijiyama Park, Minami-ku, Hiroshima 732-0815, Japan

**Keywords:** atomic bomb survivor, radiation risk, late health effects, transgenerational effects

## Abstract

This year marks the 80th anniversary of the atomic bombings of Hiroshima and Nagasaki. Over the past eight decades, large-scale cohort studies of atomic bomb survivors and their offspring conducted by the Radiation Effects Research Foundation and its predecessor, the Atomic Bomb Casualty Commission, have provided critical insights into the long-term health effects of radiation exposure. Key findings include early identification of radiation-associated leukemia, as well as excess risks of all solid cancers combined, and most individual cancer sites. Observed radiation dose–response relationships have generally followed a linear-quadratic model for leukemia and a linear model for all solid cancers. Recent findings indicating possible upward curvature in the dose–response for all solid cancers may reflect underlying heterogeneity in factors related to individual cancer sites and should be explored further. Generally, younger age at exposure, lower attained age, and female sex appear to show greater radiation sensitivity for all solid cancers combined but results differ by individual cancer site. Recent studies have also identified potential radiation-related excesses for non-cancer diseases such as cataracts, various circulatory diseases, and others. Studies of heritable effects on the offspring of exposed atomic bomb survivors, in contrast, have shown no elevated risk to date in offspring from parental radiation exposure, either at the molecular or disease level. With the cooperation of the atomic bomb survivors and their families, Radiation Effects Research Foundation’s research will continue to play a crucial role in informing the health of survivors, their families, and global radiation protection in the decades to come.

## Graphical Abstract

**Graphical Abstract:**
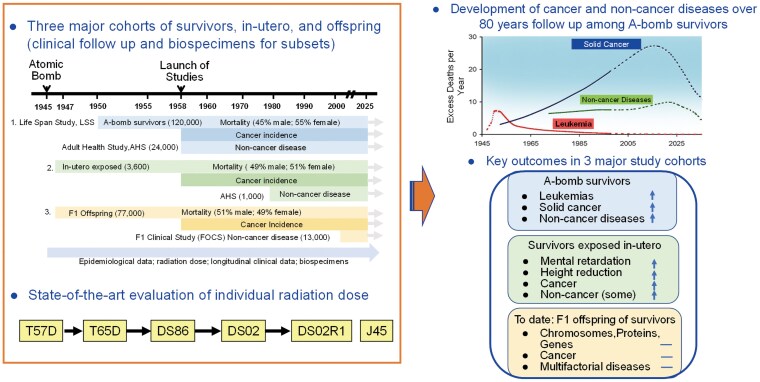

## 1. Introduction

On 6 August 1945, the first atomic bomb used on a civilian population in human history was dropped on Hiroshima, followed by the bombing of Nagasaki on August 9. According to reports from both cities, ∼140 000 people in Hiroshima [1] and 74 000 in Nagasaki [2] had died by the end of December 1945. Addressing widespread concerns regarding the potential for long-term effects, the Atomic Bomb Casualty Commission (ABCC) was established in 1947 by the U.S. National Academy of Sciences (NAS), with funding from the government of the United States. Research to elucidate the medical and biological effects of atomic bomb radiation in humans was initiated with a base in Hiroshima. The ABCC Nagasaki Laboratory was established in 1948, and in the same year, Japan’s National Institute of Health, under the Ministry of Health and Welfare, officially joined the research efforts [3]. In 1975, based on an agreement between the U.S. and Japanese governments, ABCC was reorganized into the Radiation Effects Research Foundation (RERF), a legally recognized nonprofit foundation under Japanese law [4]. RERF inherited ABCC’s research mission, but operates with equal funding from both governments, is managed by a binational Board of Directors, and guided by a Board of Councilors and Scientific Advisory Committee, each composed of an equal number of U.S. and Japanese experts [5]. This year marks the 50th anniversary of RERF’s establishment.

RERF’s lifelong cohort studies of atomic bomb survivors, *in utero*-exposed individuals, and children of survivors have contributed not only to the healthcare, medical treatment, and welfare of atomic bomb survivors and their families but also to universal knowledge elucidating the effects of radiation exposure on human health [6–8]. Research findings obtained through the long-term cooperation of atomic bomb survivors and their families have provided fundamental scientific data for the global radiation protection system established by the United Nations Scientific Committee on the Effects of Atomic Radiation (UNSCEAR), International Commission on Radiological Protection, International Atomic Energy Agency, and others [9, 10]. On the occasion of the 80th anniversary of the bombings, we provide a brief overview of RERF’s major findings and outline future directions for the institution.

## 2. Overview of RERF cohorts

**Figure 1:**
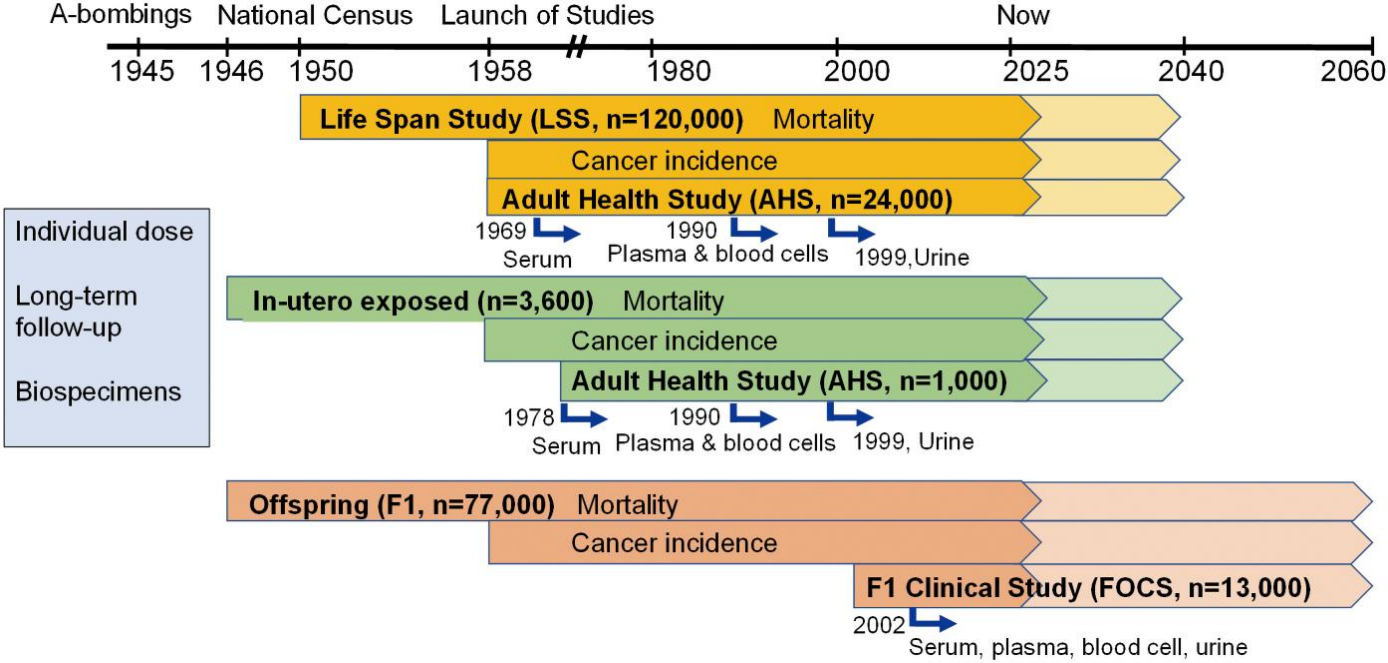
Long-term cohorts of survivors and their children maintained at the Radiation Effects Research Foundation (RERF).

RERF maintains five high-quality cohort studies of survivors and their offspring to study radiation-related risks of cancer and non-cancer disease (Fig. 1, Table 1). Studies of the survivors include the Life Span Study (LSS) of ∼120 000 individuals, including atomic bomb survivors addressing radiation-related risk of mortality (all causes) and cancer incidence; the Adult Health Study (AHS), a subsample of ∼25 000 LSS participants enrolled in a longitudinal clinical program to evaluate radiation-related non-cancer outcomes, and a small cohort (n∼3600) to assess health effects in A-bomb survivors exposed *in utero*. Potential hereditary effects of radiation are assessed in studies of children of the survivors, including the F1 Study addressing potential effects of parental radiation on mortality in ∼77 000 individuals addressing potential effects of parental radiation on mortality in offspring of A-bomb survivors, and the F1 Offspring Clinical Study (FOCS, n∼13 000), evaluating potential health effects of parental radiation on offspring in a clinical subset. The unique importance of RERF’s cohorts stems from the combination of their large size, wide range of exposure levels, inclusion of all ages at exposure, and long-standing, high-quality follow-up of disease outcomes [6–8]. Individual radiation doses for survivors are calculated through a robust dosimetry program coordinated by an international dosimetry working group [11–13]. In addition, clinical examinations and the collection of serial biosamples from cohort participants enable molecular research that will allow deeper insight into the pathogenesis and molecular basis of radiation-related health conditions.

**Table 1:** Status of RERF cohorts of atomic-bomb survivors and their children.

Cohort	Number of members	Percent of cohort alive	Average age (years)	As of date
Life Span Study (LSS) of A-bomb survivors	120 321	21%	83	Dec. 2020
F_1_ Study of Offspring of A-bomb Survivors	76 819	86%	63	Dec. 2020
*In Utero* Study	3354	78%	74	Dec. 2020
Adult Health Study, AHS	25 379	20%	86	Feb. 2025

### 2.1. The LSS and AHS of atomic bomb survivors

LSS cohort of atomic bomb survivors represents the longest running radiation epidemiology study in the world, distinguished by its large scale and exceptionally low rate of loss to follow-up (only 0.07% of the study population were lost to follow-up). The unique nature of LSS provides critical advantages for radiation risk estimation, including: (i) the inclusion of people exposed to radiation of both sexes and at various ages, allowing investigation of risk differences by sex and age; (ii) accurate estimation of individual radiation dose to study participants, enabling quantitative assessment of risk, including form of the dose-response relationship; and (iii) general population exposure, minimizing concerns of possible biases observed in medical or occupational radiation settings [6–8]. Nevertheless, the LSS has unavoidable limitations. Since the study began in 1950, data from the first 5 years after the bombing (1945–50) are not considered. Additionally, information on cancer incidence is available only post-1958, when cancer registries were established in both Hiroshima and Nagasaki.

**Figure 2:**
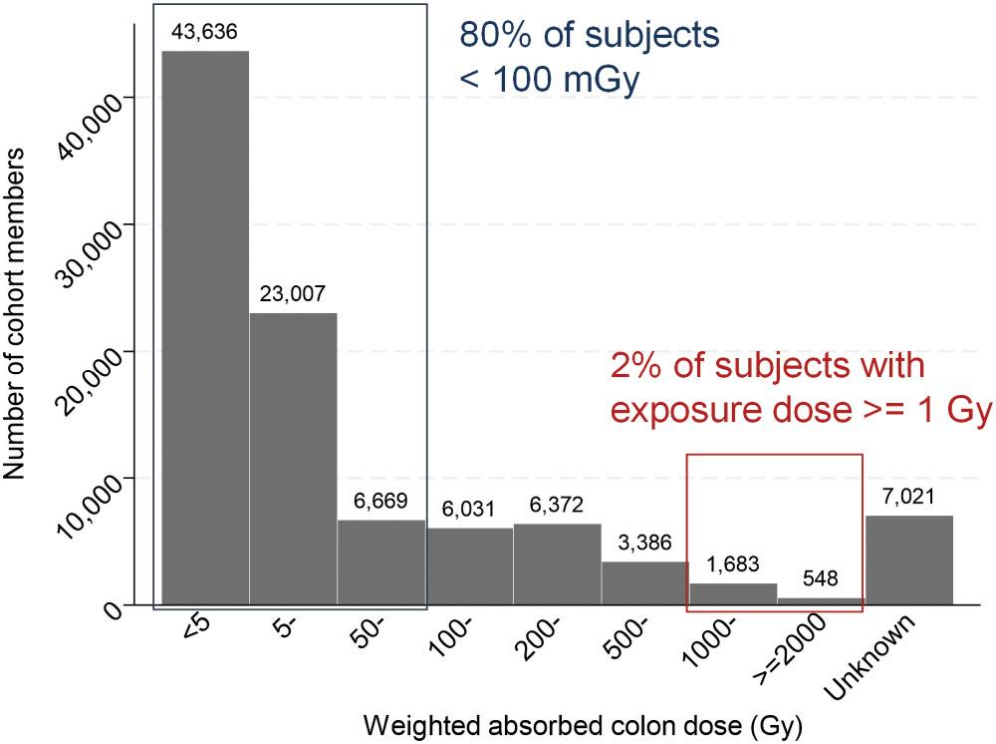
Distribution of DS02R1 weighted absorbed colon radiation dose in the life span study cohort. *Note*. DS02R1, Dosimetry System 2002 Revision 1. Weight absorbed colon dose was calculated as the sum of gamma ray dose plus 10 times the neutron dose.

Early research at ABCC comprised a series of short-term separate studies without uniformity or continuity. However, following an external committee recommendation to establish a long-term study with a fixed cohort [14], the LSS was initiated in 1958 based on the Atomic Bomb Survivors Survey conducted during the 1950 national census. Of the ∼284 000 survivors identified in the survey, the LSS cohort consists of the following four groups selected from a base population of ∼200 000 people living in Hiroshima or Nagasaki at the time of the survey, as well as those who were not in the two cities at the time of the atomic bombings [5]: Group 1—survivors exposed within 2000 meters from the hypocenter (close-proximity survivors); Group 2—survivors exposed at distances of 2000–2499 meters from the hypocenter (outer close-proximity survivors); Group 3—survivors exposed at distances of 2500–9999 meters (distant survivors), and Group 4—individuals who were >10 000 meters from the hypocenter, or not in the city at the time of the bombing (non-exposed group). Groups 3 and 4 were matched to Group 1 by city, sex, and age at exposure, with an equal number of individuals randomly selected. In 1968, an additional 9530 individuals who had been exposed within 2500 meters were added, followed by the addition of 11 409 distant survivors of Nagasaki in 1980. As a result, the total number of LSS cohort members reached 120 321. As shown in Fig. 2, the dose distribution in the LSS population is highly skewed, with 80% of cohort members having an estimated dose of less than 100 mGy and 2% of all members having a dose of 1 Gy or more.

A second cohort, the AHS, was also established in 1958 to investigate the relationship between radiation and the incidence of non-cancer disease and general health conditions. AHS cohort members were selected from the 4 groups of LSS members classified based on distance from the hypocenter and presence or absence of acute symptoms. All survivors exposed within 2000 meters who exhibited acute symptoms (Group 1) were included in the AHS. Participants (Group 2: exposed within 2000 meters who did not exhibit acute symptoms, Group 3: exposed 3000–3499 m and Group 4: same with Group 4 of LSS) from the other three LSS groups were selected in numbers approximately equal to Group 1, matched for city, sex, and age at exposure. The AHS cohort has undergone several expansions including the inclusion of approximately 1000 subjects from the *In Utero* Clinical Study since then, with 25 379 participants in total.

As of the most recent follow up data (through to the end of 2020), 21% of the initial LSS was still alive, with an average age of 83 years for living members (Table 1). Of those still alive, 67% were exposed at age 10 years or younger, an age group known to have a higher sensitivity to radiation, and thus particularly important to continue surveying for long-term health risks.

### 2.2. *In utero* exposed cohort of atomic bomb survivors

The *in utero* exposed cohort was initially established as two separate studies: the Mortality Study Cohort and the Clinical Study Cohort [5]. The Mortality Study Cohort consisted of 2802 children born between the date of the atomic bombings and the end of May 1946. Mothers of cohort members were identified through a central file of all survivors known to the ABCC to form the following five groups of *in utero* survivors: (i) all survivors exposed *in utero* whose mothers were within 1500 meters of the hypocenters, and four groups of individuals matched by information source, city, sex, and birth month to the first group, whose mothers were located within (i) 1500–1999 meters; (ii) 2000–2999 meters, (iii) 3000–9999 meters, and (iv) individuals who were not exposed *in utero.*

The *In Utero* Clinical Study Cohort included individuals born between the day of the bombings and the end of April 1946 who were alive and residing in Hiroshima or Nagasaki as of 1 October 1950. This cohort consisted of three groups based on the mother’s distance from the hypocenter: Group 1 (exposed within 2000 meters), Group 2 (exposed at 3000–4999 meters), and Group 3 (non-exposed individuals—located beyond 10 000 meters). Groups 2 and 3 were matched by city, sex, and birth month with Group 1. The total number of participants in this cohort was 1,606, of whom 1021 were later incorporated into the AHS in 1978. A combined total of 3638 *in utero* exposed individuals is currently being followed in mortality studies, reflecting some overlap between the two cohorts.

### 2.3. F1 and FOCS cohorts of offspring of atomic bomb survivors

Evaluating potential hereditary genetic effects of parental radiation exposure on the unexposed children of atomic bomb survivors has been a key focus of research since the establishment of the ABCC. The F1 cohort comprises a total of 76 814 individuals, including children of survivors who were conceived after parental exposure: 53 519 individuals born between May 1946 and December 1958 identified through interviews with pregnant women or from birth certificates submitted in Hiroshima and Nagasaki, and children of LSS cohort members born between 1959 and 1984 [5]. Participants of the former group were selected based on parental exposure distance: (i) children with at least one parent exposed within 2000 meters of the hypocenter, (ii) children with at least one parent exposed between 2000 and 9999 meters, and (iii) children of non-exposed parents (10 000 meters or beyond).

In 2002, the FOCS was initiated in an F1 subset of ∼13 000 individuals to investigate potential radiation-associated heritable effects of non-cancer multifactorial diseases: hypertension, hypercholesterolemia, diabetes mellitus, angina pectoris, myocardial infarction, and stroke [5].

## 3. Collection and storage of biological samples

Every 2 years, AHS conducts a health examination during which biological samples are collected from study participants for research purposes. Health examination and sample collection for FOCS participants occurs every 4 years. Serum provided by AHS participants has been collected and stored since 1969, plasma since 1990, and urine since 1999. Samples have been collected from *in utero* participants beginning in 1978, and FOCS health examinations starting in 2002. As of December 2024, these biological specimens now number over 2.3 million stored samples including blood and urine samples. Collection of these serial samples at each health examination, along with results of the health examinations, has facilitated the conduct of research elucidating the pathogenesis and molecular basis of conditions related to radiation exposure.

## 4. Estimation of radiation dose

Accurate estimates of individual dose are critical to the assessment of radiation-related risk [11–13]. RERF’s detailed dosimetry system is described more fully in this issue. Briefly, exposure to atomic bomb radiation consists of two components: “initial radiation” emitted at the moment of detonation, followed by “residual radiation.” The atomic bomb nuclear explosions generated various types of ionizing radiation, including neutrons, alpha particles, beta particles, and gamma rays. However, alpha and beta particles did not reach ground level in significant amounts. As a result, initial radiation exposure of the residents of Hiroshima and Nagasaki was primarily due to neutrons and gamma rays, causing external exposure to the human body within a very short period (from a few seconds to tens of seconds). Residual radiation in the two cities largely comprised induced radiation resulting from neutron activation of buildings and soil, and radioactive particles which were dispersed into the atmosphere by the nuclear explosion.

Factors evaluated to estimate initial radiation dose included: (i) radiation output of the bomb, (ii) attenuation by distance from the hypocenter, (iii) shielding effects from terrain (e.g. hills) and construction, and (iv) attenuation within the body after radiation reaches the body surface. In the Ichiban Project, field measurements conducted at the U.S. Nevada Test Site in 1957, 1958, and 1962, measured the radiation output of nuclear explosions, atmospheric attenuation, and shielding effects of Japanese-style wooden houses [15]. Field surveys and interviews were conducted in Hiroshima and Nagasaki to determine individual conditions at the time of the bombing. Information collected for individual dose estimation included detailed location at the time of the bombing, presence and characteristics of shielding materials, location inside buildings (e.g. whether on the first or second floor, distance from walls or windows), and body posture (e.g. sitting, standing, orientation relative to the bomb).

RERF’s dosimetry system, developed by a multidisciplinary team of international experts, has undergone multiple improvements to enhance its accuracy. The first dose assessment, T57D (Tentative 1957 Doses, published in 1957), was a tentative estimate rarely used in analyses. Subsequently, T65D (Tentative 1965 Doses) was introduced in 1965, followed by the establishment of the first comprehensive dose assessment system, DS86 (Dosimetry System 1986), which was used for the first detailed epidemiological risk estimates of solid cancer [16]. With advancements in computational technology, DS86 enabled the simulation of neutron and gamma-ray transport while accounting for shielding conditions to produce individualized dose estimates for 15 organs, incorporating information on exposure location, shielding conditions, and body posture at the time of bombing. Further refinements led to the development of DS02 (Dosimetry System 2002), completed in 2002 [17], and more recently, DS02R1 doses updated based on information from a 2017 review of survivors’ mapped locations and shielding conditions. The DS02R1 doses have been used for the most recently published set of analyses, including radiation-related cancer incidence risks for follow-up through to 2009 [12]. For evaluation of health effects, organ doses corresponding to the target disease are used. The analysis employs a weighted absorbed dose, calculated by adding the estimated gamma-ray dose to the weighted neutron dose (i.e. gamma dose + neutron dose × 10). RERF’s international dosimetry working group is currently finalizing new dose estimates, which will provide doses for 81 distinct organs or organ sites.

Dose estimation by the RERF does not include exposure from residual radiation. Evaluation of exposure from residual radiation, which is composed of radioactive fallout and induced radiation from neutron-activated radionuclides, requires detailed information, including individual behavioral records over time after exposure, the environmental dynamics of radioactive particles (fallout), contamination levels of food and water and individual intake patterns, and estimates of inhaled radioactive substances from the atmosphere. Collecting such information shortly after the bombing was not practical given the difficult circumstances. Consequently, RERF’s risk estimates rely exclusively on initial radiation, for which individual dose estimates are available. Although information regarding exposure to residual radiation is limited, the following estimates have been made: (i) according to DS86 estimates of “induced radiation,” a 12-hour stay on the day following the A-bombing at a location 500 meters from the hypocenter resulted in ∼15 mSv in Hiroshima and ∼3 mSv in Nagasaki [16]; (ii) the estimated residual radiation exposure dose for 99 members of the Kahoku Unit engaged in relief activities in Hiroshima from August 7 to 13 was a maximum of approximately 100 mSv and an average of ∼13 mSv [5]; and (iii) an internal exposure survey conducted using whole-body counters on residents of the Nishiyama district in Nagasaki (where the amount of radioactive fallout was estimated to be the highest) estimated cumulative doses over 40 years to be ∼100 µSv for men and 80 µSv for women [18]. Based on these assessments, exposure to residual radiation is believed to be significantly lower than exposure to initial radiation [5]. DS86 evaluates residual radiation exposure as falling within the margin of error for the estimated initial radiation exposure. Moreover, since residual radiation exposure is unlikely to have varied significantly for different groups within the initial radiation exposure distribution, excluding residual radiation is unlikely to significantly impact risk estimates [19].

## 5. Long-term health effects of radiation

### 5.1. Malignant disease

Based on observations from LSS cohort updates of mortality (solid cancer and non-cancer outcomes through to 1997; leukemia through to 1990), the first radiation-related excess of death from malignant disease observed in atomic bomb survivors was leukemia, which appeared ∼2 years after exposure, peaked 6–8 years after the bombing, and rapidly declined thereafter [20]. However, excess risk of leukemia death has persisted even beyond 50 years after exposure. Among leukemia subtypes, acute myeloid leukemia (AML), chronic myeloid leukemia (CML), and acute lymphoblastic leukemia (ALL) show significant increases in radiation-related risk [21, 22]. Although some findings suggest a possible radiation-related increase in chronic lymphocytic leukemia, the limited number of cases makes it difficult to draw definitive conclusions. A significant radiation dose–response relationship has also been observed for myelodysplastic syndrome, which has been registered as a malignant disease beginning around 2000 [23]. Radiation-related risk of ALL and CML declined sharply over time, with almost no cases observed after 1960. In contrast, excess radiation-related risk of AML and myelodysplastic syndrome appear to have continued even after 2000 [23].

Analyses in both the LSS and AHS indicate no clear association between radiation and risk of multiple myeloma [24].

**Figure 3:**
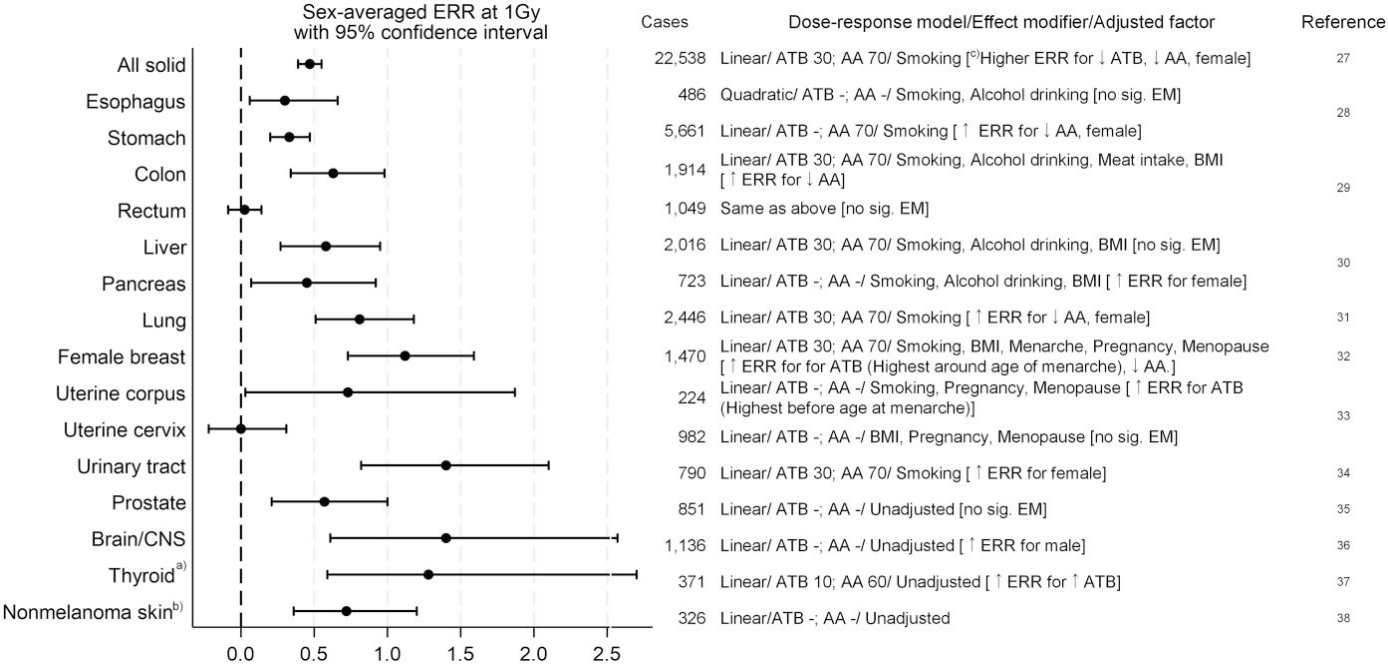
Radiation risks for incidence of major cancer sites, follow-up 1958–2009. *Note*. (a) Analysis for the period of 1958–2005, (b) Analysis for the period of 1958–1996, (c) Statistically significant effect modification (EM) at *P* < 0.05. ERR, excess relative risk; ATB, age at the time of bombing; AA, attained age; BMI, body mass index.

In contrast to leukemia, excess solid cancer death due to radiation began to be observed ∼10 years after the bombing. Overviews of radiation-related incidence and mortality risks for solid cancers have been described in comprehensive analyses by Thompson *et al*. in 1994 [25], Preston *et al*. in 2007 [26], Ozasa *et al*. in 2012 [27], and Grant *et al*. in 2017 [28]. Individual cancers of the esophagus, stomach, colon, liver, gallbladder, lung, bladder, skin (excluding melanoma), brain/central nervous system, thyroid, and breast were found to be significantly associated with radiation exposure in the cancer incidence follow up from 1958 to 1998 [26] and/or the mortality study with follow up from 1950 to 2003 [27]. Summary risks from the most recent set of studies of cancer incidence risks followed up from 1959 to 2009 are presented in Fig. 3 [28–39]; these results are described in detail by Brenner *et al*. in this issue. Briefly, radiation-related incidence risks were significantly elevated for the previously reported cancer sites, as well as for cancers of the pancreas [31], uterus corpus [34], and prostate [36]. In contrast, no significant increase in radiation-related risk was observed for oral cancers other than salivary gland, or cancers of the rectum, biliary tract, larynx, kidney parenchyma, uterine cervix, or ovary. Although variations in the magnitude of risk were observed across cancer sites, overlapping confidence intervals for many sites make the exact magnitude of variation unclear.

RERF collaborates with city hospitals in Hiroshima and Nagasaki to collect and systematically review pathological specimens of cancer cases among LSS members to enable detailed studies of risk by histological subtype based on standardized criteria accounting for changing disease classification or advances in diagnostic technology during LSS’s long follow-up period. Results of analyses for cancers of the liver, salivary gland, skin, ovaries, thyroid, lungs, lymphoid system, and central nervous system have indicated particular sensitivity to radiation for certain tumor subtypes, such as mucoepidermoid tumors within salivary cancers [40] and precursor cell neoplasms within lymphoid neoplasms [41], as well as patterns of risk by subtypes of CNS tumors [42] diagnosed by pathologists. Histology-based analyses for cancers of the breast, uterus, and bone/soft tissue sarcoma are ongoing.

Genetic analysis of pathological tumor tissues from atomic bomb survivors, on the other hand, have revealed increased *p53* point mutations in hepatocellular carcinoma [43] and *PTCH1* deletions in basal cell carcinoma [44] following radiation exposure. In thyroid cancer, *BRAF* point mutations predominated at lower doses, while fusion genes such as *RET*, *ALK* were more frequent at higher doses [45, 46]. These genetic mutations provide insights into the mechanisms of radiation-induced carcinogenesis.

#### 5.1.1. Dose–response relationship

Previous analyses of LSS cancer incidence (1958–1997) and mortality (1950–2003) indicated a linear dose response for all solid cancers combined in both males and females over the full dose range, with an estimated sex-averaged excess relative risk per gray (ERR/Gy) of 0.47 for incidence and 0.42 for mortality for individuals exposed to 1 Gy at age 30 years, with attained age 70 years [26, 27]. However, mortality analyses by Ozasa *et al*. [27] indicated an upward curvature when the dose range is limited to <2 Gy, and the most recent analyses of solid cancer mortality and incidence indicated possible upward curvature in dose–response that was not limited to either sex, or to incidence or mortality data [47]. However, the underlying reasons for the curvature remain unclear, and likely reflect heterogeneity in factors including site-specific baseline rates and models, form of high dose adjustment, and choice of representative organ dose. Although the linear model described most sites adequately, other dose-response functions provided a better fit in some instances, such as the quadratic model for esophageal cancer [29] and the linear-threshold model with a threshold at 0.63 Gy for basal cell carcinoma [39] (Fig. 3).

Analyses of radiation-related risk of leukemia incidence (1950–2001) indicate a linear-quadratic relationship for AML and a linear relationship for ALL and CML. The relationship for all leukemia cases combined was strongly influenced by AML, which constituted most cases, resulting in an overall linear-quadratic dose-response [22].

#### 5.1.2. Modifiers of radiation-related risk of cancer

Individual susceptibility to radiation can be influenced by both intrinsic (e.g. age and sex) and extrinsic factors (e.g. use of tobacco/alcohol, viral exposure). In general, individuals of younger age, lower attained age, and female sex, appear to be more susceptible to radiation-related cancer, although patterns of risk vary by individual cancer site [26–28] (Brenner, 2025, this special issue). The sex-averaged ERR for all solid cancers combined decreases with increasing age at exposure, and declines with increasing attained age. A similar pattern has been observed for leukemia. However, age effects vary by cancer site—for example, thyroid cancer risk is strongly influenced by age at exposure, with higher risk in younger individuals [48], but radiation-related lung cancer risk increases with increasing age at exposure [32]. Breast cancer and uterus corpus cancer, on the other hand, demonstrate distinct patterns that appear to be associated with hormonal factors: with highest excess breast cancer risk for individuals exposed around the time of menarche [33], and the highest radiation-related endometrial cancer risk with exposure just before the onset of puberty [34]. Overall radiation-related risk for solid cancers combined is higher in females than in males, but again this differs based on individual site, with higher radiation-related risks observed for males in brain/CNS cancers, and no clear effect of modification by sex for colon cancer. External factors that modify radiation risk have been observed for lung cancer in relation to smoking [32] and for liver cancer in relation to HBV infection [49].

### 5.2. Non-cancer diseases

Radiation-related risk of non-cancer diseases in atomic bomb survivors has been assessed through follow-up of LSS mortality and AHS clinical outcome data. The LSS mortality data from 1950 and 2003 indicated significant increases in radiation-related mortality due to circulatory, respiratory, and digestive diseases [27, 50]. More detailed analysis of LSS heart disease mortality from 1950 to 2008 found significant radiation-related excesses of hypertensive heart disease, rheumatic heart disease, and heart failure but showed no significant associations between radiation exposure and ischemic heart disease and myocardial infarction [51]. Incidence data from AHS Report 8 (follow-up from 1958 to 1998) indicated significantly increased radiation-related risk among atomic bomb for cataracts, uterine myoma, thyroid disorders, chronic liver disease and cirrhosis, hypertension [52], and another report has demonstrated an increased prevalence of hyperparathyroidism [53]. Results for diabetes have been inconsistent—with a relationship observed in data from Hiroshima but not in Nagasaki, suggesting the need for further examination of these results [54]. To date, no clear associations have been found between radiation exposure and the risk of neurocognitive function in old age among those exposed childhood or *in utero* [55, 56].

### 5.3. *In utero* exposure

Although the cohort size is small and interpretation is complicated by potential confounding factors, studies of *in utero* radiation exposure in atomic bomb survivors have indicated some evidence of adverse effects. Radiation exposure during 8–15 weeks and 16–25 weeks of gestation was associated with increased risk of intellectual disability, lower intelligence quotient, and poorer academic performance. However, no significant effects were observed in individuals exposed before 8 weeks or after 26 weeks of gestation [57]. Additionally, investigations of *in utero* exposure indicated increased risk of small head sizes in individuals exposed before 15 weeks of gestation, but no significant increase in those exposed at 16 weeks or later [58, 59]. These data suggest possible windows of susceptibility to exposure during specific stages of brain development, which could differ depending on specific outcome. *In utero* radiation exposure was also found to significantly impair physical development, particularly with respect to height reduction, with no clear relationship between age of gestation and radiation sensitivity [60].

The most recent follow-up of solid cancers following *in utero* radiation exposure suggests that solid cancer incidence risk is increased, possibly at a magnitude lower than the excess risk of solid cancers observed in LSS survivors exposed as children (<6 years) [61]. To date, excess risk of leukemia has not been analysed in those exposed *in utero* because of the small cohort size and the rare occurrence of leukemia.

With respect to mortality, analyses of *in utero* atomic bomb survivors followed up from 1950 to 2012 indicated a significant increase in risk associated with maternal uterine dose for non-cancer mortality among males, as well as for cancer, non-cancer disease, and external causes of death mortality among females. After adjustment for small head size, low birth weight, and loss of parents—factors significantly associated with radiation dose—estimated risks for mortality other than cancer in females were attenuated and were no longer statistically significant, while increased risk for cancer mortality in females persisted [62].

Interpretation of the *in utero* results requires careful consideration of small sample size, intermediate factors, and potential confounding by factors such as sanitary conditions and poor nutritional status resulting from the atomic bomb's aftermath—further follow-up of these cohorts will provide additional clarity regarding cancer and non-cancer effects.

### 5.4. Studies of potential heritability of radiation effects

The possibility that parental exposure to radiation could result in adverse health effects to their children has been a key concern for survivors, their families, and the scientific community in the aftermath of the atomic bombing. Potential hereditary effects have thus been a strong focus of research at RERF since the days of ABCC. Early attempts to address this question included large-scale surveys of birth defects [63, 64], examination of the sex ratio [64] of children of atomic bomb survivors conceived after the bombing, as well as differences in chromosomal aberrations [65], serum proteins [66, 67], and DNA mutations [68–71], with each analysis using state-of-art techniques available at the time. Although these data have been reanalysed in recent years [72], it is difficult to draw clear conclusions due to issues such as a lack of information on the damage caused by the atomic bombings to their family and household economic conditions, which are strongly related to radiation exposure levels. To date, these studies have detected no evidence of molecular effects of parental radiation exposure on their offspring.

At the disease level, incidence of cancer, mortality from cancer and other diseases, and prevalence of non-cancer diseases have been evaluated through ongoing follow-up of the F1 and FOCS cohorts, respectively. So far, analyses of cancer incidence (1958–1997) [73], cancer, and non-cancer disease mortality (birth to 2009) [74], and prevalence of multifactorial diseases including hypertension, hypercholesterolemia, diabetes, angina, myocardial infarction, and stroke [75] have indicated no significant increase in any of these diseases related to parental radiation exposure among the offspring of atomic bomb survivors. However, as the children of atomic bomb survivors are now reaching ages where cancer and lifestyle-related diseases occur more frequently, continued long-term follow-up and careful investigation remain essential.

## 6. Future directions

Moving into the next decade, research conducted at RERF will continue to provide more detailed scientific evidence on the health effects of radiation on atomic bomb survivors and their children. This body of research will remain a crucial source of fundamental data for radiation protection, playing a vital role in establishing national and international radiation protection standards through various policy-making and regulatory bodies, including the International Commission on Radiological Protection, UNSCEAR, and others. Significant questions remain to be answered from continued follow-up of RERF’s epidemiologic cohorts, including the identification of emerging radiation-related risks of cancer and non-cancer diseases, and more precise characterization of risks that have already been identified. As radiation protection increasingly considers the possibility of individualized protection, it will be crucial to better understand the shape of dose–response relationships for overall solid cancers and for specific cancer sites (and subsites), as well as effect modification by intrinsic factors (e.g. age, sex) and extrinsic factors (e.g. use of smoking and alcohol). The same is true for non-cancer outcomes. Addressing ongoing concerns regarding possible transgenerational effects of radiation, follow-up of the F1 and FOCS cohorts for the lifetimes of the children of the atomic bomb survivors will be essential.

Underpinning RERF’s estimates of radiation-related disease risk are dose estimates provided by the international dosimetry working group [11–13]. With advances in technology, particularly much higher-fidelity models of the human body (“phantoms”) and more sophisticated analytical techniques, RERF is currently finalizing new estimates that will provide doses for 81 distinct organs or organ sites, compared to the previous 15 organ estimates. While the previous computational stylized phantoms included only three androgynous models (infant, child, and adult), the sophisticated voxel phantoms (J45) will include detailed organ topography and composition, six age categories, sex-specific phantoms for adolescents and adults, and phantoms for pregnant female and the fetus—allowing for more targeted analyses, and improved assessment of *in utero* exposure, and effect modification by factors such as age and sex.

Supplementing the epidemiological data, RERF’s ongoing collection of serial biological specimens places the institution in a unique position to address questions of individual susceptibility as well as underlying mechanisms of radiation-related disease through analysis and integration of data from serially collected biospecimens. The availability of baseline and subsequent samples from the same individuals presents a tremendous opportunity for risk prediction and disease prevention, elucidating genetic and epigenetic mechanisms of radiation-associated disease, and identifying radiation biomarkers and/or “signatures.” Already, RERF is launching projects leveraging state-of-the-art molecular biology and computational techniques to evaluate questions such as the potential for *de novo* mutations in children of exposed parents; further examining the relationships between radiation and processes such as clonal hematopoiesis [76, 77], alterations related to T lymphocyte immunosenescence [78, 79], and persistent inflammation [80] that have been observed among atomic bomb survivors, and integration of clinical images with epidemiological data to predict radiation-related disease. A planned study of whole genome sequencing study of blood samples collected since 1985 from “Trios” (mother-father-child), will allow for a more precise characterization of potential hereditary effects than has been possible in the past. With rapid advances in technology continuing to arise over the next decades, we expect that these integrated studies of high-quality long-standing epidemiologic and clinical data with serial collections of biospecimens will provide key insights into the nature of radiation risk in humans.

RERF’s research has been made possible through the cooperation of many atomic bomb survivors and their children, to whom we express our deepest gratitude. Honoring their invaluable partnership, we remain committed to continuing these investigations ensuring that their cooperation continues to benefit themselves and their offspring, as well as science and humanity.

## Data Availability

The data associated with this review is available from the corresponding authors on a reasonable request.
